# Stabilization and Dispersion of OSA Starch-Coated Titania Nanoparticles in Kappa-Carrageenan-Based Solution

**DOI:** 10.3390/nano12091519

**Published:** 2022-04-30

**Authors:** Xingyu Chen, Kai Wu, Sili Zeng, Da Chen, Lingyun Yao, Shiqing Song, Huatian Wang, Min Sun, Tao Feng

**Affiliations:** 1 School of Perfume and Aroma Technology, Shanghai Institute of Technology, Shanghai 201418, China; chen2864578472@163.com (X.C.); 15738285855@163.com (K.W.); lyyao@sit.edu.cn (L.Y.); sshqingg@163.com (S.S.); wanghuatian@sit.edu.cn (H.W.); sunmin@sit.edu.cn (M.S.); 2 Shanghai Beukay Cosmetics Co., Ltd., Shanghai 201418, China; zengsl@beukay.com; 3 Department of Food Science and Technology, The Ohio State University, Columbus, OH 43210, USA; chen.10570@osu.edu

**Keywords:** titania pigment, OSA starch, K-CG, spray drying

## Abstract

Titania is a white pigment used widely in papermaking, paints and cosmetic industries. Dispersion and stabilization of high concentration titania in water-based system remains a great bottleneck in industry nowadays, because aggregation of titania nanoparticles results in severe adverse effects to gloss, opacity, tint strength, color distribution and storage stability of end products. Because kappa-carrageenan (κ-CG) has excellent rheological properties such as emulsification, gelation, stability and so on, it has the ability to form gel and increase the viscosity of aqueous solution. In this work, Octenyl succinic anhydride (OSA) starch was utilized as wall material to encapsulate titania pigments using electrostatic spray drying processing. Transmission electron microscopy (TEM) showed that titania pigments were coated by OSA starch, with a final form of nanoparticle. Accelerating stability test found that around 60% OSA starch–titania particles were stably dispersed in κ-CG-based solution. All materials used in this work were natural ingredient, which would be preferred by cosmetic industry and consumers. The technique used in the present study could potentially be extended to other pigments for similar purpose.

## 1. Introduction

Titania, a white pigment, is widely used in paint, plastics and color cosmetic industries to bring appealing color for many products. It has been reported that five million tons of titania was produced annually in the global [1]. The pigment (rutile) displays a higher refractive index, excellent whitening and covering capacity compared to that of anatase, zinc sulfide and other commonly used white pigments [2]. Nevertheless, due to its higher density and electrostatic interaction, dispersion and suspension of high content of titania pigment can be hardly achieved in water-based system [3], because the pigment tends to aggregate and leads to serious side effects on the gloss, opacity, coating durability, tint strength, color distribution and storage stability [4] of final products. Therefore, many artificial dispersants such as polyacrylic acid or polyacrylamides copolymer were used to disperse titania [5]. Some other researchers modified polyacrylamides including homo and copolymers with carboxylate and/or hydroxyl groups as dispersants to improve the dispersion properties [6]. However, cosmetic regulations on restricting organic solvents as well as safety concern decreased their usage [1]. In recent years, polysaccharide-based nanoparticles have been widely used in biomedical products for enhancing biocompatibility and stability of various bioactive components [7]. For cosmetics, coating titania with polysaccharides may provide an alternative approach for preparation of water-based titania pigment.

Octenyl succinic anhydride (OSA) starch, obtained from hydrophobic esterification of native starch molecules, is widely utilized to protect bioactive components or natural ingredients against harsh conditions [7,8,9]. In a previous study, OSA starch and chitosan blend was used for protection of β-carotene [8]. To date, OSA starch has been employed as wall material to enhance stability and availability of various active substances by formation of microspheres after freeze drying [10] or spray drying. However, the application of OSA starch to coat inorganic material as titania pigment has rarely been investigated.

Carrageenan (CG) is a sulphated linear polysaccharide, mainly obtained from certain red seaweeds of the Rhodophyceae class [11,12]. It consists of D-galactose residues linked alternately of 3-linked-β-D-galactopyranose or 4-linked-β-D-galactopyranose units. According to the degree of substitution of the free hydroxyl groups, CG are generally categorized into six basic forms, i.e., kappa (κ)-, iota (τ)-, lambda (λ)-, mu (μ)-, nu (ν)-, and theta (θ)-carrageenan [13]. Of which, kappa-carrageenan (κ-CG) possesses outstanding properties such as low cost, non-toxicity, biocompatibility, biodegradability, and low immunogenicity [14,15]. κ-CG has been used as emulsifier, thickener and stabilizer, and a low-concentrated κ-CG solution was generally applied to dairy products as shape fixing agent [16]. Pandey et al. [17] found that the synthesis of silver nanoparticles (AgNPs) nanocatalyst by using high-molecular-weight κ-carrageenan. The synthesized nanocatalyst exhibited high catalytic degradation and mineralization of industrially important organic dyes such as Rhodamine B, and methylene blue, with a degradation efficiency of ~100% in a very short interval. Fan et al. [18] prepared novel Salecan/κ-carrageenan composites HGs (CHGs) by physical approaches and conducted comprehensive rheological and thermal studies on their distinctive properties. It is proved that they have ideal anti-freezing ability, enhanced thermostability, good injectability, self-recovery, and other rheological properties. One study showed that the percentage of –CG in the substrate (–CG–HPMC) was up to 40%, which could well control drug release (salbutamol sulfate and chlorpheniramine maleate) at an early stage, and the release curve was nearly linear [19,20].

In this work, a new method for stabilization and dispersion of titania pigment was developed. The agents involved in stabilization and dispersion of titania are natural in origin and biodegradable, which is considered as a better alternative for the cosmetic industry and consumers. Titania pigment was firstly coated with OSA starch through spray drying to form nanoparticles. Thereafter, the nanoparticle was dissolved in a low-concentrated κ-CG-based solution. In addition, rheology, transmission electron microscopy (TEM) and accelerated stability tests were used to assess the effect of OSA starch on pigment particle dispersion. The proposed method would not only improve the dispersion and suspension of titania pigment, but may also be applicable to other pigments in food and cosmetic products for similar purpose.

## 2. Materials and Methods

### 2.1. Materials

The rutile sample used throughout this investigation was purchased from Shandong Xiangxin Chemical Co., Ltd. (Shanxi, China), with a TiO_2_ content of 97 to 99%. Rutile was an aluminate- and zirconia-coated aluminum-doped with a density of 4.1 g/cm^3^. OSA starch (A.R.) was purchased from Ingredion China Ltd. (Shanghai, China). κ-CG (A.R.) was obtained from Shanghai Brilliant Gum Co., Ltd. (Shanghai, China), with a mass-average molecular weight (Mw) of 2.3 × 10^5^ Da. Purified water (G.R.) was commercially obtained from Shenzhen Watsons distilled water Co., Ltd. (Shenzhen, China).

### 2.2. Preparation of OSA Starch—Titania Pigment Nanoparticle (OTP)

OSA starch (100 g) was added into 900 mL purified water, and then heated at 60 °C to make an OSA starch solution (10 wt.%). An amount of 500 g of titania pigment was added into OSA starch solution and stirred for 5 min to make a suspension. The suspension was loaded to a spray dryer (Model-110EH, Microfluidics Corporation, Westwood, MA, USA) and operated at a 100 MPa pump pressure with 3 processing cycles, an inlet temperature of 40 °C and an outlet temperature of 60 °C [21]. The dried powder was named as starch—titania pigment nanoparticle (OTP).

### 2.3. Characterization of OTP

#### 2.3.1. Morphology and Structure Analysis

Transmission electron microscopy (TEM; JEM-ARM200F, JEOL, Freising, Germany) was applied to observe the morphology of titania pigment before and after encapsulation by OSA starch [22]. OTP or non-processed titania pigment (0.01 g) was dispersed in 9.9 mL water and sonicated for 2 min. An aliquot of the dispersion was added onto a carbon film-coated copper grids (300 mesh) and dried quickly with flush air prior to be imaged at an acceleration voltage of 150 kV.

#### 2.3.2. TG Analysis and DSC Analysis

TG (Thermogravimetric) analysis was conducted as described previously with modifications [23]. Titania pigment, OSA starch and OTP samples were scanned using a Q5000 thermogravimetric analyzer (TA Instruments, New Castle, DE, USA) in the temperature range of 0–500 °C with a heating rate of 10 ℃/min under a controlled N_2_ gas atmosphere with a flow rate of 30 mL/min.

Differential scanning calorimetry (DSC) was conducted to evaluate thermality of OTP using a DSC Q2000 instrument (TA Instruments, New Castle, DE, USA). The sample (15 mg) was placed in DSC pans and heated from 0 to 160 °C at a 10 °C/min rate [24].

### 2.4. Dispersion of OTP in κ-CG-Based Solution or Water

κ-CG (2 g) was dissolved in 198 mL water to prepare a κ-CG-based solution (1 wt.%). OTP of 40, 45, 50, 55 g were mixed with 60, 55, 50 and 45 g κ-CG-based solution or water. Amounts of 40, 45, 50, and 55 wt.% of OTP mixture were obtained for stability test.

### 2.5. Quality and Stability Test of Titania Pigment Slurry

#### 2.5.1. Contact Angle and Surface Tension

Contact angle (θ) and surface tension (σ) are critical parameters for hydrophilicity between liquid to a surface and liquid molecular cohesion. Contact angle (θ) refers to the angle from the solid–liquid interface through the liquid interior to the gas-liquid interface at the junction of the solid, liquid, and gas phases, and is a measure of wettability. Surface tension is the force that acts on the surface of a liquid that reduce the surface area. Cohesion allows liquids to resist to tensile stress, while adsorption allows liquids to adhere to other objects. OTP was dispersed in a κ-CG-based solution and purified water as samples accordingly, surface tension and contact angle of samples were measured mainly according to Konstantin Ludanov’s research with proper modifications [25]. They were carried out in a closed room at 25 °C to avoid any vibration with theta flex. For contact angle analysis, pig skin was utilized to simulate human skin. Drop rate was set as 2 μL/s; drop volume was set at 1 μL for surface tension analysis. The measurements were done in triplicate and the average results were shown (Theta Flex, Espo, Finland).

#### 2.5.2. Particle Size Distribution

Particle size of titania pigment and OTP were measured by Mastersizer 3000 (Malvern Panalytical, Malvern, UK) according to Wu et al. with minor changes [26]. An amount of 0.01 g of Titania pigment and OTP were dispersed in sample tank accordingly.

#### 2.5.3. Zeta Potential

OTP (5 g) was dispersed in 45 mL water or κ-CG-based solution. Put it into the sample tank for ζ measurement using a Zetasizer (Nano-ZSP, Malvern Instruments, Worcestershire, UK) as the work [27]. The surface zeta potential attachment uses tracer particles to measure electroosmosis near the sample surface and calculate the zeta potential of the surface.

#### 2.5.4. Viscosity

OTP (40 g, 45g, 50 g, 55 g, and 60 g) was dispersed in 60 mL κ-CG-based solution and purified water, respectively. Dynamic viscosity was measured with number 1 rotator at a 60 rpm with Viscometer (NDJ/5S, Qingdao, China).

#### 2.5.5. Accelerated Stability Tests

Dispersions of OTP in κ-CG-based solution or water were adjusted to pH 7. They were loaded to a dispersion Analyzer (LUMiSizer dispersion Analyzer, Berlin, Germany). The parameters were set as follows: processing duration was 250 min; temperature was 25 °C; rotation speed was 3000 rpm [28]. Instability index from 0 to 1 corresponding to extremely stable to highly unstable dispersion was thus acquired [29].

### 2.6. Statistical Analysis

All the experiments were carried out in triplicate. Data were analyzed using a one-way analysis of variance (ANOVA). All the graphs were processed with an Origin 9.0 (Origin Lab Co. Ltd., Northampton, MA, USA).

## 3. Results and Discussion

### 3.1. Microstructure of Titania Pigment and OTP

**Figure 1:**
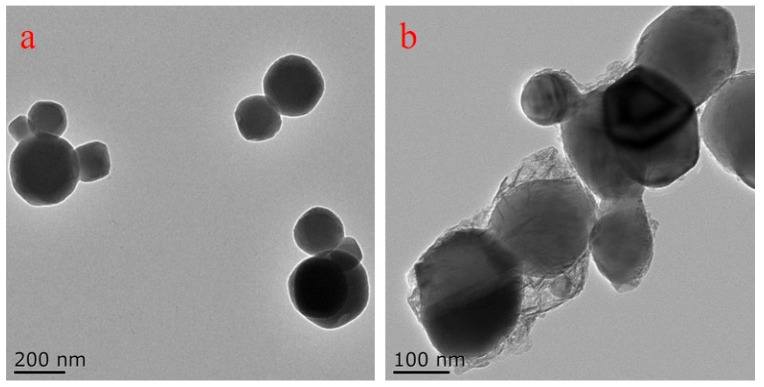
Typical TEM images of titania pigment (**a**) and OTP (**b**).

As shown in Figure 1a, the particle size of titania pigments was ranged mainly from 50 to 300 nm with a round shape. The darkness of the pigment was nearly the same across the particles, indicating a homogenous structure. After encapsulation with OSA, a transparent layer with less density was observed surrounding the titania pigment. The layer showed irregular shape with varied thickness (Figure 1b).

An adsorption layer was also observed in ethylhydroxy ethylcellulose-stabilized TiO_2_ and Fe_2_O_3_ aqueous dispersions [30]. Due to distinct chemical nature, the ability of electron penetration differs, thus leading to different transparency on the TEM images. After OSA starch encapsulation, some of the formed OTP particles were still assembled due to Van der Waals forces, which would result in instability for titania pigment dispersion [23].

### 3.2. Particle Size Distribution

**Figure 2:**
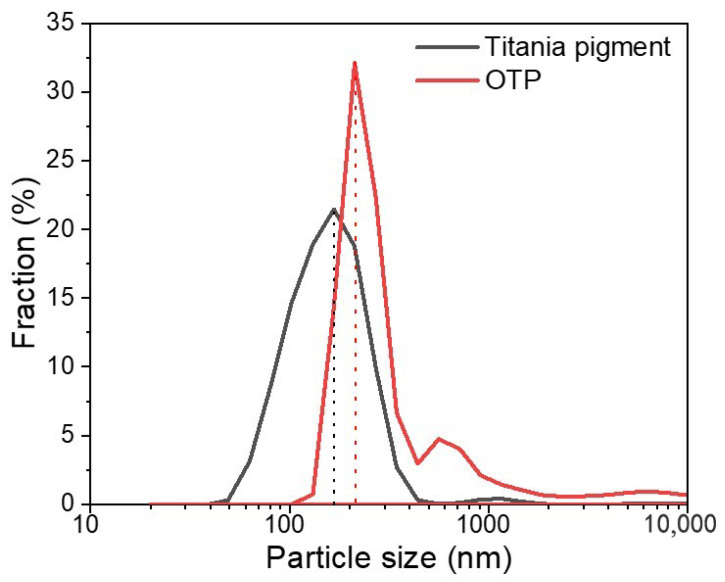
Particle size distribution of titania pigment and OTP in deionized water.

Particle size distribution (PSD) indicates the mass frequency and uniformity of distribution of particles. As shown in Figure 2, the PSD of titania pigments was ranged from 40 to 500 nm, whereas those of OTP had a wider range (100–700 nm). The mean size of titania pigments was around 150 nm, and it increased to 200 nm in OTP. In general, finer titania pigments were modified into coarse particles after being coated with OSA starch. The PSD of particles was critical for particle dispersion stability in colloids system [31]. The nanosized level of the OTP would be helpful to maintain good stability in water-based system [32].

### 3.3. TG and DSC Analysis

**Figure 3:**
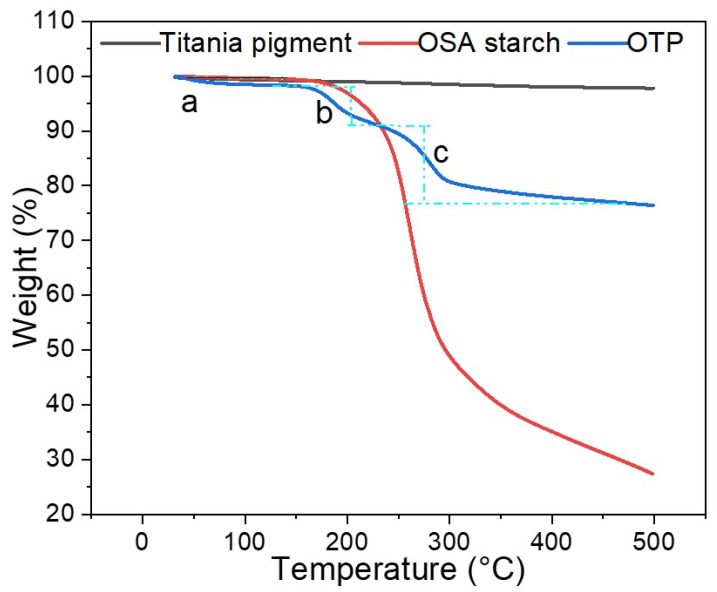
Thermogravimetric analysis (TGA) of titania pigment, OSA starch and OTP.

Figure 3 showed the weight loss of titania pigment, OSA starch and OTP under tested TG analysis condition (in the temperature range of 0 to 500 °C). A continuous and slight weight loss for titania was observed after 100 °C, which may be attributed to degradation of impurities from titania pigment. It has been reported previously that titania pigment was heat resistant material [1]. There was about 1% of weight loss in total within 100 °C. After 100 °C, the loss was about 2% of total weight. OSA starch is mainly carbohydrate with the water absorption capacity [7], the weight loss within 100 °C could be due to the loss of moisture (Figure 3). From 200 to 300 °C, hydroxy structures of OSA starch started to degrade [33], resulting in a rapid reduction in weight (Figure 3). Further increases in temperature also led to a large amount of weight loss, and at the end of heating, only 25% of the original weight remained. At temperatures above 200 °C, OSA starch would reach the degrading point and the decomposition of the macromolecules occurred [34]. For OTP, the weight loss can be divided into three main stages according to Figure 3. Initially, moisture was evaporated from particle and caused slight weight loss below 100 °C (stage a in Figure 3). When the temperature was higher, OSA starch layer began to lose weight (stage b). After 200 °C (stage c), the OSA starch layer degraded and OTP particles lost weight drastically. After 400 °C, it could be predicted that mainly naked titania pigment was left, and no more weight loss was observed. OTP mainly comprised OSA starch and titania pigments, in addition to some sticky moisture. According to the blue line (OTP), after 450 °C, around 75% of weight were remained. According to the black line (titania pigment), after 450 °C, around 2–3% of moisture evaporated. At 100 °C, OTP lost about 1% of moisture. Compared to titania, it could be predicted that OSA starch layers contributes to 22% of the total weight.

**Figure 4:**
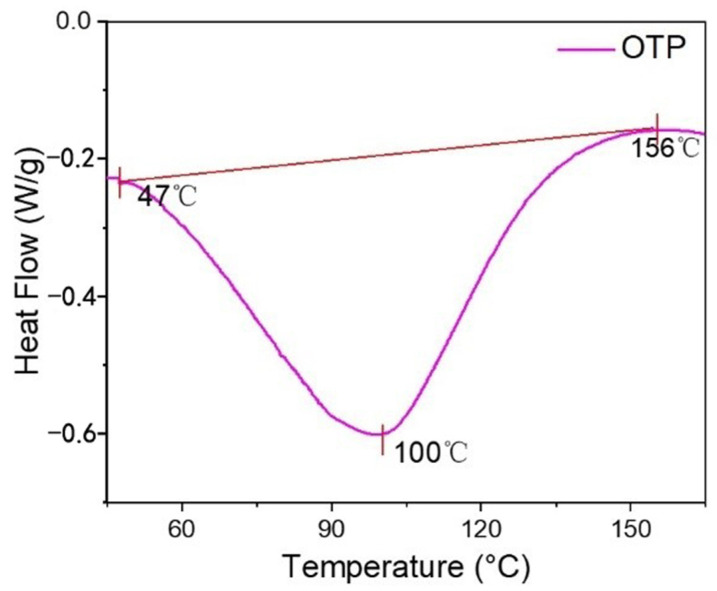
Differential scanning calorimetry (DSC) analysis of OTP.

The calorimetric thermograms of OTP was depicted as a graph of heat flow (W/g) versus temperature (°C) and shown in Figure 4. The onset temperature, glass transition temperature (T_g_), and offset temperature were around 47 °C, 100 °C and 156 °C, respectively [35]. Generally, the onset temperature was accepted as melting temperature at which OTP particle started to change its physical form at 47 °C. This was attributed to the chain unfolding or re-arrangement of the OSA starch layer. At 100 °C, OTP reached the glass transition temperature, indicating that the starch coated on titania started to transform from glass state to a higher elastic state until 156 °C. Below 47 °C, OTP was remained in a stable shape, whereas its quality was seriously damaged after 100 °C.

### 3.4. Stability and Quality of Titania Pigment Slurry

#### 3.4.1. Contact Angle and Surface Tension

**Figure 5:**
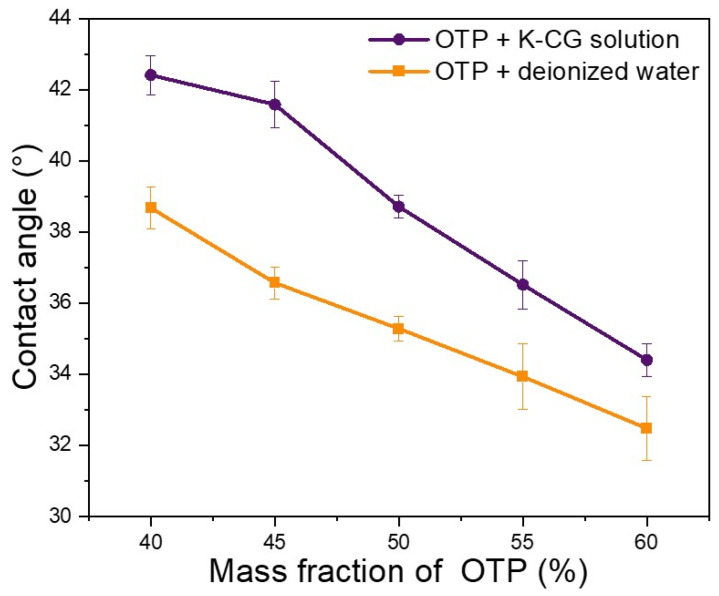
Contact angle in dispersing OTP in water and K-CG solution.

Contact angle analysis is an indicator of both interfacial energy between solution and surface, whereas surface tension reflects the liquid molecular cohesion [25]. Hydrophilic refers to a molecule with a polar group that has a high affinity to water. Here, pig skin was used as simulate of human skin, different contents of OTP dispersed in a κ-CG aqueous solution or water were used as samples for contact angle analysis. As shown in Figure 5, the contact angle decreased with the increase in OTP content in a κ-CG solution or water. In addition, compared to pure water, OTP in κ-CG had larger contact angles at the same content, indicating a higher surface hydrophilicity of a κ-CG-based solution might be easily adhered to simulated skin.

**Figure 6:**
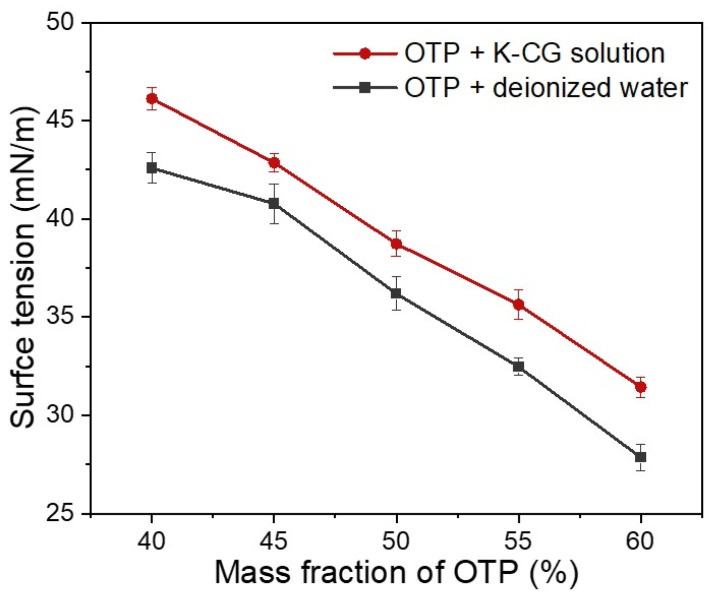
Surface tension in dispersing OTP in water and κ-CG solution.

Figure 6 demonstrated the surface tension of OTP dispersed in both a κ-CG solution and water [36]. With the increasing content of OTP, their surface tension decreased drastically (Figure 6). Similarly to contact angle analysis, OTP dispersed in a κ-CG solution showed a higher surface tension than that in pure water. The increased contact angle meant a higher interfacial energy between solution and the surface. This change may be caused by the presence of κ-CG as compared with pure water suspension. As κ-CG is often used as a thickener, it can produce a weak network in water-based solution [11]. The contact angle and surface tension analysis demonstrated that OTP dispersed in a κ-CG-based solution showed a higher hydrophilicity and liquid molecular cohesion, suggesting that a κ-CG aqueous solution was more suitable for OTP stabilization and dispersion.

#### 3.4.2. Viscosity Measurement in OTP Suspension

**Figure 7:**
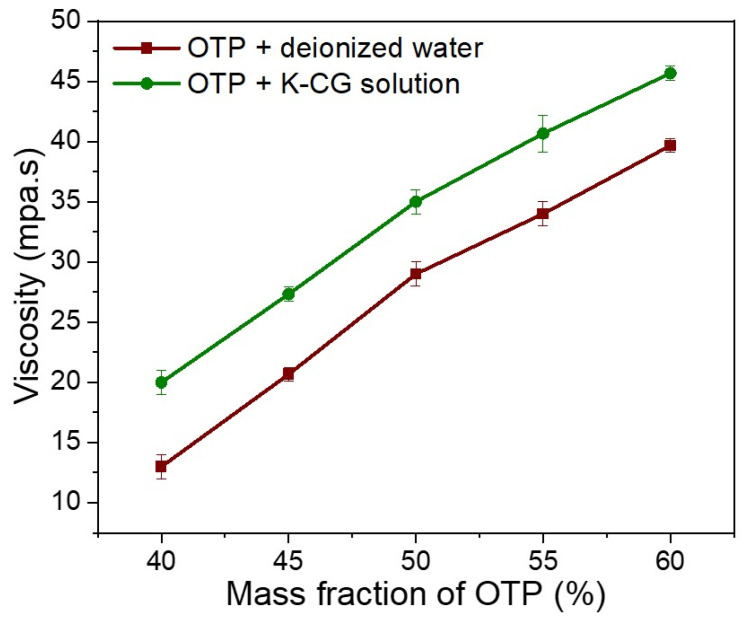
Viscosity in dispersing OTP in deionized water and K-CG solution.

The viscosity increased sharply with the increase in OTP contents in both dispersion. In κ-CG dispersion, the highest viscosity of 46.3 MPa·s was observed at the maximum OTP content (60%, *w/w*). Compared to the dispersion in water, OTP dispersed in a κ-CG aqueous solution showed a higher viscosity at the same content of OTP (Figure 7).

Viscosity, contact angle and surface tension are three vital indicators for stabilization and dispersion of OTP nanoparticle slurry. Compare with dispersion in pure water, three parameters were enhanced due to the functional properties of κ-CG (Figure 5, Figure 6 and Figure 7). κ-CG has been widely utilized in pharmaceutical, food, and cosmetical industries owing to its thickening, stabilizing and gelling properties [13,16]. The obtained data in this work suggested that a κ-CG aqueous solution was successfully employed for stabilization and dispersion to OSA starch-coated titania pigment through regulating the viscosity, contact angle and surface tension of the suspended coating nanoparticles.

#### 3.4.3. Zeta Potentials

**Figure 8:**
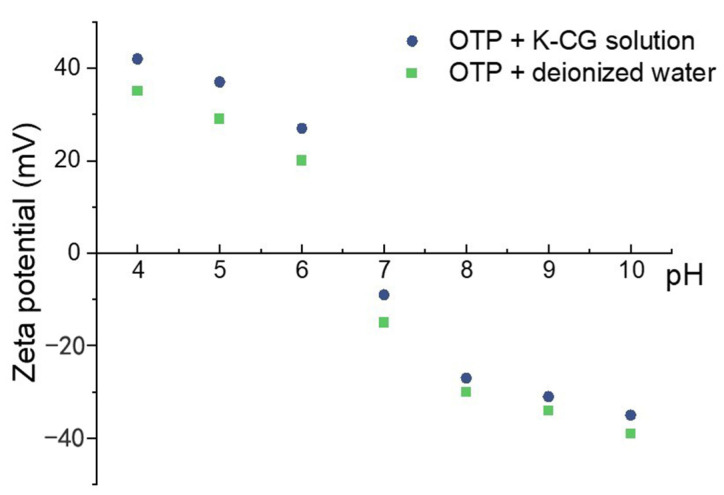
Zeta potential of dispersing OTP in water and K-CG solution.

Zeta potentials (ζ) were detected to evaluate the stability of colloidal dispersions, while higher ζ value representing better stability [37]. Figure 8 showed a comparison of ζ values between OTP dispersed in a κ-CG solution and deionized water under various pH. The ζ value of the nanoparticles were negative above pH 7 and changed to positive with reduction in pH to acidic conditions (Figure 8). At pH 4, 5, and 6, dispersing OTP in a κ-CG solution presented a higher ζ value than that in deionized water, suggesting the solution would be more stable. On the contrary, at pH 7, 8, 9, and 10, dispersing OTP in deionized water presented higher ζ value and better stability. As κ-CG is an anionic carbohydrate [11], dispersing OTP in κ-CG would change the isoelectric point and therefore keep the stability of the pigment suspension when pH at 4, 5, and 6 [38].

#### 3.4.4. Accelerated Stability Tests

**Figure 9:**
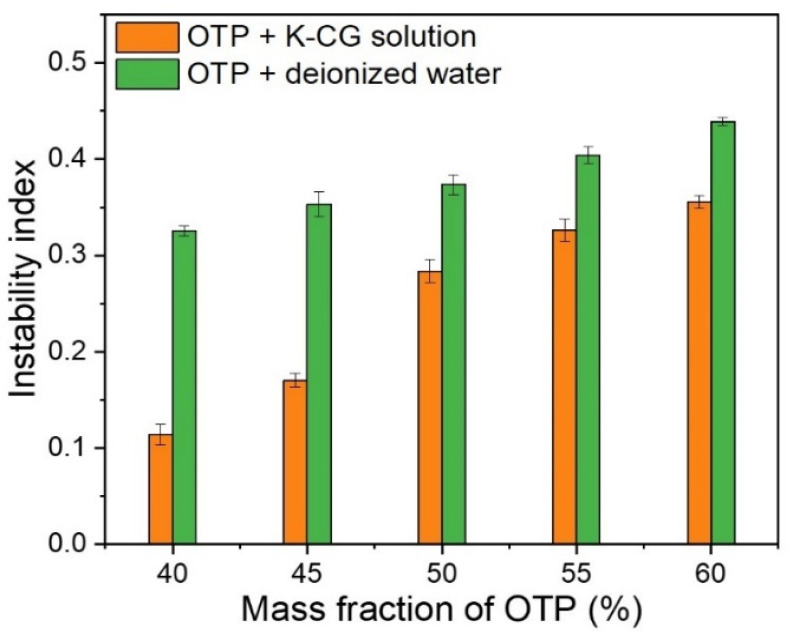
Instability index in dispersing OTP in water and K-CG solution.

As shown in Figure 9, the instability index of colloids system was plotted against the OTP mass fraction. With the increase in OTP content, colloids system became much more unstable (i.e., relatively higher instability index value). For the same content of OTP, dispersing in a κ-CG solution showed a lower instability index than that in deionized water (Figure 9). High content of pigment particles generally represented wider particle size distribution, which was often taken as the contributor to instability of slurry [3,5]. Therefore, higher OTP mass fraction would result in an increase in instability for both a κ-CG-based solution and deionized water. Dispersing OTP in a κ-CG solution revealed relative low instability index (0.34) at the maximum tested content (60%). In consideration of the high pigment content and stability in cosmetic products, OSA starch-coated titania nanoparticles in a κ-CG solution was suggested to be an efficient and useful method for dispersing pigments in an environmentally friendly colloidal system.

Thereafter, OTP were dispersed in a κ-CG-based solution or deionized water. Dispersing OTP in a κ-CG-based solution presented better stability compared to dispersing OTP in deionized water, which guaranteed a longer shelf life, as shown in Figure 8 and Figure 9. Dispersing OTP in a κ-CG-based solution illustrated comparably higher viscosity, contact angle and surface tension according to Figure 5, Figure 6 and Figure 7. This was due to κ-CG, which was generally considered as a popular thickening agent, emulsifier [16]. In general, this novel formulation was considered to improve the quality and stability of titania pigment slurry.

### 3.5. Study on the Mechanism of Particle Suspension

The gelation capacity of carrageenan depends on the structure of its main disaccharide units, determined in particular by the presence of 4-bonded D-galactose residues in the form of 3,6-anhydrous derivatives, the number of sulfate groups per 1 carrageenan unit and their position in the molecule [39]. In addition, the gelling properties of the polysaccharides increased with the increase in 3,6-anhydrogalactose content in them [40]. Instability is due to aggregation or less charge as well as particle size. Aggregation was driven by non-covalent interactions. κ-CG does not have any effect on those.

**Figure 10:**
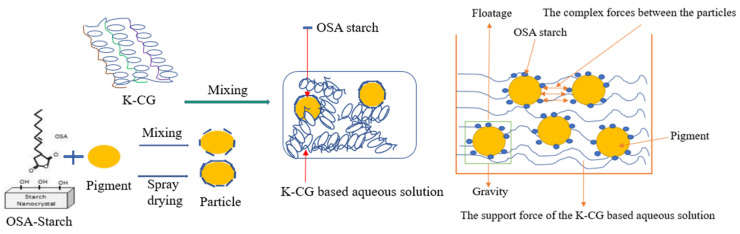
Schematic diagram of process flow and particle suspension mechanism.

In this work, titania pigment was firstly coated with OSA starch through spray drying to form nanoparticles. Thereafter, the nanoparticle was dissolved in a low-concentrated κ-CG-based solution. According to Figure 10, the gravity indicating the force due to the pigment’s own mass is related to the size of the pigment. Floatage is related to the difference in density between the pigment and the κ-CG-based aqueous solution. The support force of the κ-CG-based aqueous solution, indicating the support effect of the weak network of the κ-CG-based aqueous solution itself on the pigment. Electrostatic interactions are related to the type of ion molecules, and electrostatic forces involve the attraction and accumulation of oppositely charged ions on a charged surface [41]. Adsorption of an anionic polymer onto a negatively charged surface may occur if, for example, the attractive force of hydrogen bonding exceeds the electrostatic repulsion. Chemical interactions occur at the surface sites of the polymeric dispersant groups and the pigment particles and may form covalent or ionic bonds through reactions between the polymers [42]. The dispersant functions to assist in wetting out the pigment, prevent settling, and stabilize the pigments by ensuring pigment separation in the dispersion. Vehicle is essentially the liquid that the particulate medium is dispersed in. By the encapsulation of OSA starch and the gelation property of carrageenan, the pigment particles do not coagulate, forming a homogeneous and stable solution.

## 4. Conclusions

OSA starch was applied to encapsulate titania nanoparticles and formed OTP after spray drying. The obtained OTP particles were characterized by the TEM, TG and DSC analysis, the results indicated that OTP has been successfully prepared with particle size ranging from 100 to 700 nm and revealed excellent thermal stability with temperatures ranging from 0 to 100 °C. Furthermore, stabilization and dispersion property of OTP in a κ-CG solution and deionized water was investigated. Contact angle and surface tension analysis manifested that high content OTP in κ-CG displayed better hydrophilic and liquid molecular cohesion as compared to those in water. Accelerating stability test revealed that around 60 wt.% of OTP were dispersed and stably suspended in the κ-CG-based solution. However, the adsorption mechanisms and thickness control of OSA starch layer on titania pigment were still unclear, and more efforts are required for expanding its further utilization in the future. In conclusion, OSA starch-coated titania nanoparticles in a κ-CG solution was suggested to be an efficient and useful method for dispersing pigments in an environmentally friendly colloidal system.

## Data Availability

The data presented in this study are available on request from the corresponding author.
